# Influence of Crack Width in Alternating Tension–Compression Regimes on Crack-Bridging Behaviour and Degradation of PVA Microfibres Embedded in Cement-Based Matrix

**DOI:** 10.3390/ma13184189

**Published:** 2020-09-21

**Authors:** Majid Ranjbarian, Xiaomeng Ma, Viktor Mechtcherine

**Affiliations:** Institute of Construction Materials, TU Dresden, 01062 Dresden, Germany; steffima24@gmail.com (X.M.); mechtcherine@tu-dresden.de (V.M.)

**Keywords:** cement-based composites, fibre reinforcement, PVA microfiber, interface properties, fibre pull-out test, crack-bridging characteristics, fatigue behaviour, cyclic tension-compression loading, crack width, FRC, SHCC, ECC

## Abstract

The use of high-performance polymeric microfibres in enhancing the ductility of cementitious composites is widespread. A vivid example is the application of strain-hardening cement-based composites (SHCCs) in the construction industry. However, there are a few challenges which need to be addressed with respect to material design. For instance, the ductility of SHCC diminishes under alternating tension–compression loading, where the fibres lose their crack-bridging capacity due to specific damage mechanisms. The damage development and its influence on crack-bridging capacity have been studied in previous works by the authors. The paper at hand focuses on the influence of crack width on the crack-bridging capacity of polymeric microfibres in conjunction with the number of cycles in an alternating tension–compression regime with different cyclic compressive force levels. It shows that bridging capacity can be markedly influenced by crack width: an increase in crack width leads to more severe damage to the fibres and thus to lower crack-bridging capacity. Then, after analysing the specimens by means of electron microscopy, a hypothesis is presented to address the effect of crack width on damage development. Finally, a simple approach is proposed for estimating the influence of different parameters on fibre degradation.

## 1. Introduction

The use of cementitious materials is repeatedly compromised due to their low tensile strength and brittleness. A promising approach for improving their ductility has been the addition of dispersed short fibres [1]. Following this approach in terms of ductility different classes of fibre-reinforced cementitious composites (FRCCs) can be realised depending on their compositions. The volume fraction of fibres exerts a major influence on the post-cracking tensile behaviour of FRCCs. With a relatively small amount of fibre, often in combination with their insufficient efficiency in a given composite, a single crack occurs under uniaxial tension, and strain-softening behaviour is a typical post-cracking tensile response. For fibre content above a critical value, usually more than one crack can form, and the tensile stress can increase up to an ultimate value with increasing deformation of the composite. This class of FRCCs is usually known as strain-hardening cementitious composites (SHCCs). Many researchers have reported their work on improving the ductility of SHCC and understanding its behaviour, e.g., [2,3,4,5,6,7,8]. However, development of a simple material model that can predict the SHCC properties by interlinking the properties on different observation levels seems still to be a serious challenge despite some progress having been made in terms of the modelling so far; see e.g., [9,10].

Great tensile strain capacity can be achieved through the formation of many fine cracks or, alternatively, a few large cracks. The latter can occur if the fibres are not uniformly distributed over the volume of the matrix, leaving the volume fraction of the fibres lower in particular regions of the specimen [11]. Although SHCCs generally demonstrate high ductility, their behaviour can change substantially with the type of loading. One of the most destructive loading scenarios concerning post-cracking tensile behaviour of SHCCs was identified by Mueller and Mechtcherine [12]. They demonstrated that after imposing inelastic deformations on SHCC, applying only a few hundred cycles in an alternating tension–compression regime, could lead to extensive damage to the crack-bridging fibres in one or several of the cracked sections and significantly reduce their ductility. The compressive load component was clearly responsible for severe damage to microfibres, which were repeatedly squeezed between the crack faces. The authors used high-performance polyvinyl alcohol (PVA) microfibres as reinforcement, which have relatively high stiffness and strength under tensile loading in the longitudinal direction due to their fibrillar structure [13]. However, low stiffness and the compressive/shear strength of the fibres caused degradation of their crack-bridging properties after repeated squeezing between the crack flanks. Ranjbarian and Mechtcherine [14] investigated this topic further at the micro-level by performing double-sided, single-fibre pull-out tests under alternating tension–compression loading and analysing the damage to the fibres microscopically after the tests. They showed that three types of damage could occur under such a loading regime: (1) defibrillation of the fibres between crack faces, (2) fibre peeling and abrasion between crack faces, and (3) superficial abrasion in the embedded part. While the last was observed on the fibres completely pulled out, one or a combination of the two former types of damage could cause “fibrillar” fracture of the fibres [15] at tensile stresses much lower than their nominal tensile strength. As demonstrated, the severity of the damage depends considerably on the level of cyclic compressive stress and number of cycles. However, there might be other parameters influencing the damage development of the fibres and its severity in such situations. As instances, the level of bridging stress in the fibre during cyclic loading and crack width are likely to belong to them.

Crack width has always been a serious concern in respect of the durability of cementitious materials. Protecting steel reinforcement against corrosion and preventing damage to concrete by the ingress of chemically harmful elements are the main motivations to keep crack width in cementitious materials as small as possible [16]. In this regard, SHCCs are very promising, since the width of uniformly distributed fine cracks is considerably smaller than in concrete without short fibres. The cracking behaviour of SHCCs and the range of attainable crack widths have been extensively studied by many researchers. This information is relevant to the choice of a suitable range of crack width for the investigation on this parameter’s influence on the crack-bridging capacity of fibres in alternating tension–compression regime.

“Residual” crack width can be measured after failure of a specimen subject to a direct tension test. In this case permanent, inelastic deformations are considered [17]. However, measuring the real-time widths of the cracks during such tension tests usually leads to higher values, which are likely to be more representative of many practical conditions. In particular, crack widths in structural elements or specimens under loading are more relevant to studies of the influence of crack width on the fibres’ bridging capacity in alternating tension–compression regimes.

Different ranges of crack width distribution have been observed on SHCCs. Li et al. [17] reported residual crack openings of below 71 µm, while the actual crack openings were estimated to be between 80 and 160 µm for the maximum tensile stresses. Lepech and Li [18] studied the water permeability of SHCC and reported that the corresponding width of cracks was approximately 60 µm at a strain of 1.5%. Crack openings close to this value were also observed in investigations by Wang and Li [19], Wagner et al. [20], and Leung [3] for SHCCs tested under monotonic tensile loading. In a recent study of SHCC with tensile strain capacity up to 10% [2], an average crack width of 200 µm was reported as well. It seems reasonable to distinguish between the average and maximum crack width. Boshoff and Adendorff [21] emphasised that the average width of cracks in SHCC is not a suitable indicator for evaluating durability. They showed that the maximum crack width could be over 100 µm at a strain of 0.7%, while the average value pointed to be around 40 µm. This finding can also be relevant for the evaluation of the crack-bridging capacity of the fibres in alternating tension–compression loading since only critically wide crack openings in a section are expected to affect the ductility of SHCC unfavourably. Van Zijl [22] also distinguished between average and maximum crack width. While the mean crack width would range between 40 to 70 µm up to strains of 3%, the maximum crack width could be from 100 to 400 µm at strains of 0.2% and 3%, respectively. Similar observations were also reported in a comparative study [23], in which different testing laboratories were involved and a reliable database was produced. It revealed that crack width up to 250 µm was possible at strains of 3% despite the average values having remained in a range from 40 to 80 µm. Under sustained loading, even larger values for maximum crack width up to several hundred micrometres have been reported [24,25,26].

This paper presents the results of an extensive investigation into the influence of crack width along with the loading parameters in cyclic tension–compression regimes on the bridging capacity of PVA microfibres embedded in a cementitious matrix. The influence of small and large crack widths along with different loading parameters such as cyclic bridging stress level in the fibre, cyclic compressive stress level acting on the fracture section of the matrix, and number of cycles have been studied with respect to the development of damage on the PVA microfibres in alternating tension–compression regime. The damage quantification approach proposed by the authors in a previous study [14] has been adapted under consideration of crack width and relevant loading parameters.

## 2. Materials

Polyvinyl alcohol (PVA) high-performance microfibres (Kuralon K-II REC15, produced by Kuraray, Japan) are used in this work along with a matrix developed previously at the TU Dresden (Germany) [12]. The properties of the PVA microfibre and the composition of the matrix are given in Table 1 and Table 2, respectively.

## 3. Test Setup

**Figure 1:**
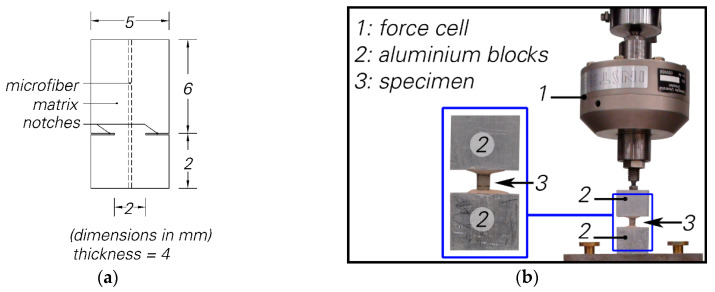
(**a**) The geometry of double-sided, single-fibre pull-out specimen, (**b**) the test setup configuration for double-sided, single fibre pull-out tests.

Figure 1a illustrates the geometry of the double-sided fibre pull-out specimen. An embedded length ratio of 3 is used to reduce the probability of two-way full debonding and slippage. The fracture section of the specimen is 8 mm^2^ (2 mm × 4 mm). As shown in Figure 1b, the specimen is glued between two aluminium blocks and, after hardening of the glue, loading begins. More information about the specimen preparation method as well as post-fracture behaviour of the specimen can be found in [27].

## 4. Experimental Programme

**Figure 2:**
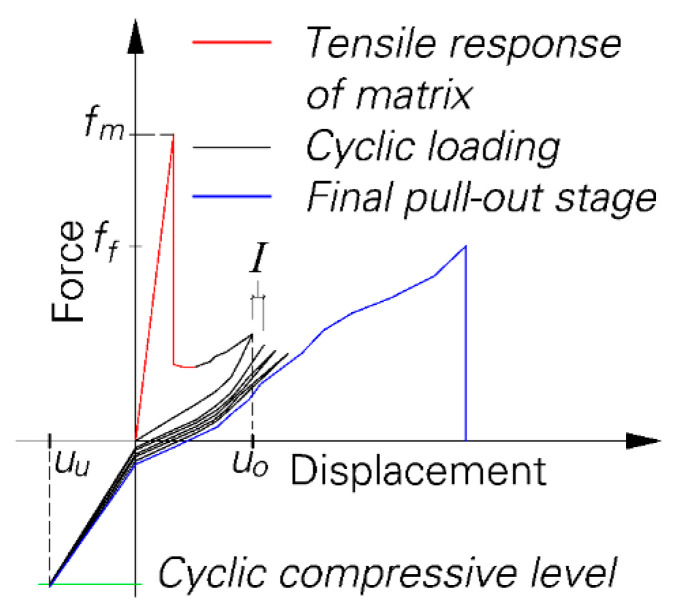
Progression of the force-displacement curve during different loading stages.

The series of specimens previously tested under quasi-static monotonic loading as presented in [14] is used as a reference for comparison. A special loading scenario is used to study the influence of crack width and loading parameters; see Figure 2. First, the specimen is pulled in a quasi-static monotonic regime until it cracks on the notches and the crack opening displacement reached a specific value of U_O_. Then, the cyclic stage begins with a predetermined number of cycles and cyclic compressive force level. In this stage, the upper and lower reversal points are displacement-controlled. For the upper reversal point a displacement increment is considered to prevent the reduction of tensile forces in the fibre during the cyclic stage. When the predetermined number of cycles is reached, the final pull-out stage begins by pulling the specimen under quasi-static monotonic loading. Loading is applied under a displacement-controlled regime with displacement rates of 0.01, 1 and 0.01 mm/s for the three stages, respectively.

The loading parameters for different testing series are given in Table 3. The testing series is divided into two main series according to crack opening displacement at the beginning of the cyclic stage; C40- and C100-, with U_O_ equalling 40 and 100 µm, respectively. At the end of the cyclic stage crack width reaches 60 and 200 µm for these two series, respectively. In previous research [28], a reduction in fibre-bridging force with an increasing number of cycles was observed due to pull-out creep and as well the creep behaviour of the fibre itself [29]. Thus, a displacement increment per cycle is considered for the upper reversal point, while its value depends on the number of cycles and the crack width in the beginning and at the end of the cyclic stage. It is also noteworthy that different bridging forces in the fibre will be attained depending on the initial and final crack width and the displacement increment in cyclic stage as well. Three different numbers of cycles–200, 1000 and 2000–are considered for the cyclic stage to study the influence of this parameter on the fibre’s damage development. Various cyclic compressive force levels are achieved by using different displacement-controlled lower reversal points. In this regard, the series is divided into three different conditions: no compressive force, moderate cyclic compressive force, and high cyclic compressive force level.

## 5. Results and Discussion

In comparing the results pull-out toughness, fibre force, and displacement at rupture are considered. For pull-out toughness, the area under the force-displacement curve from a displacement of 0.2 mm up to the end displacement of individual specimen is calculated. The results are presented for small and large crack widths separately, and finally they are compared. Since the lower reversal point is displacement-controlled in this work, the cyclic compressive forces change slightly in the cyclic stage. Hence, an average value is calculated for the maximum compressive force in the cyclic stage for each specimen. Eventually, the average compressive force is calculated for individual testing series.

Different bridging behaviours as previously explained [14] are also observed for different testing series in this study. A statistical summary of the results according to the bridging behaviour as observed is given in Table 4. For the vast majority of the specimens, fibre rupture with one-way full debonding and slippage was observed, only a few specimens exhibiting fibre rupture with two-way full debonding and slippage or complete fibre pull-out. Therefore, to evaluate the bridging properties for the different testing series, only fibre rupture with one-way full debonding and slippage was considered and the rest of the results disregarded. For many specimens tested with large crack widths, the fibre ruptured in the cyclic stage already, i.e., before reaching the final pull-out phase. Thus, zero values are taken for calculation of crack-bridging parameters for these specimens. The number of the corresponding specimens is also listed in Table 4.

### 5.1. Small Crack Width

**Figure 3:**
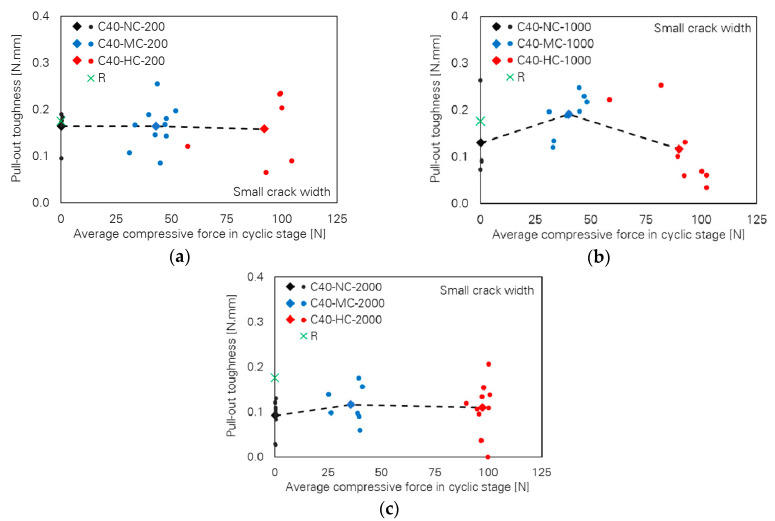
Influence of cyclic compressive force level on pull-out toughness of the specimens with small crack width; number of cycles: (**a**) 200, (**b**) 1000 and (**c**) 2000.

Figure 3 illustrates pull-out toughness as a function of the compressive force level for different numbers of loading cycles. For low numbers of cycles, pull-out toughness is almost independent of cyclic compressive force level, following a horizontal line; see Figure 3a. However, the toughness values unexpectedly increase for the series C40-MC-1000 in comparison to C40-NC-1000 and finally a decreasing tendency can be seen for C40-HC-1000; see Figure 3b. With increasing numbers of cycles, the pull-out toughness follows almost a horizontal line by increasing the compressive force level; see Figure 3c.

**Figure 4:**
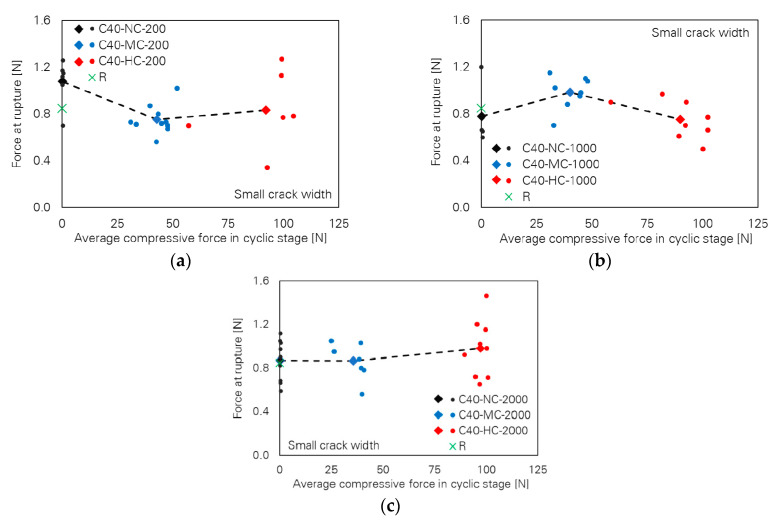
Influence of cyclic compressive force level on force at rupture of the specimens with small crack width; number of cycles: (**a**) 200, (**b**) 1000 and (**c**) 2000.

**Figure 5:**
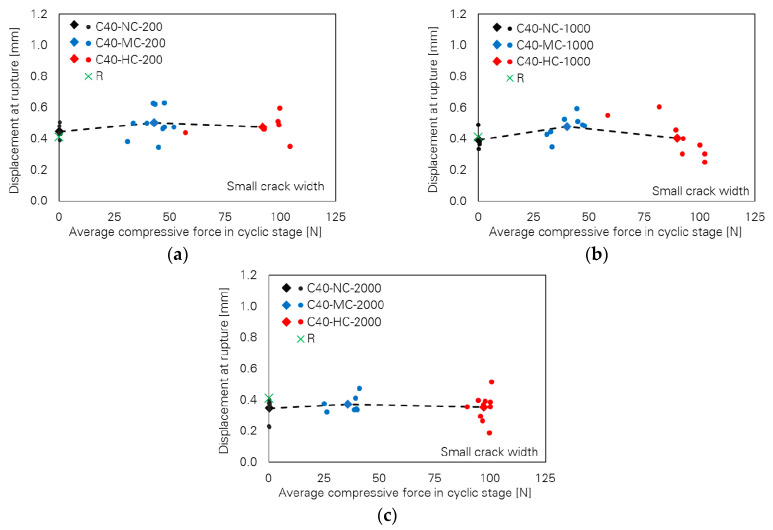
Influence of cyclic compressive force level on fibre displacement at rupture of the specimens with small crack width; number of cycles: (**a**) 200, (**b**) 1000 and (**c**) 2000.

Figure 4 shows slight differences between fibre force at rupture depending on cyclic compressive force and number of cycles. The values of force at rupture are very close, particularly for the cases with 2000 cycles; see Figure 4c. This is also the case for the values of displacement at fibre rupture as shown in Figure 5. For any number of cycles, the displacement at rupture follows a nearly straight horizontal line with increasing compressive force; the differences are negligible.

### 5.2. Large Crack Width

**Figure 6:**
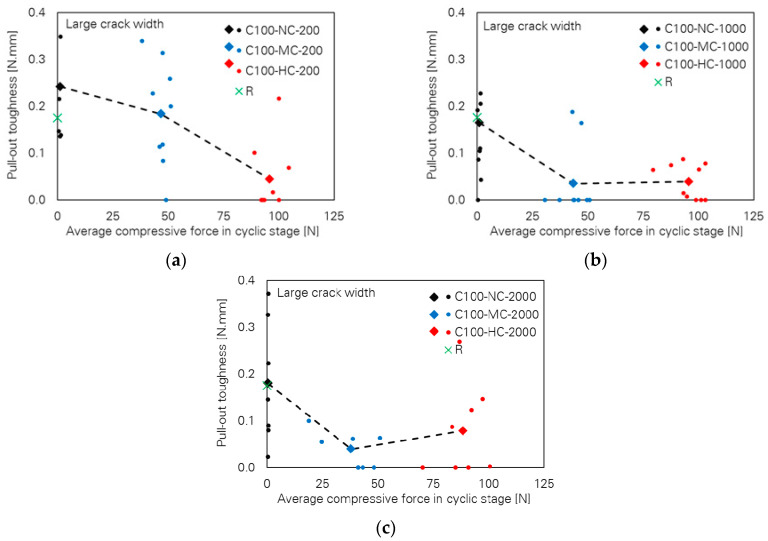
Influence of cyclic compressive force level on pull-out toughness of the specimens with large crack width; number of cycles: (**a**) 200, (**b**) 1000 and (**c**) 2000.

**Figure 7:**
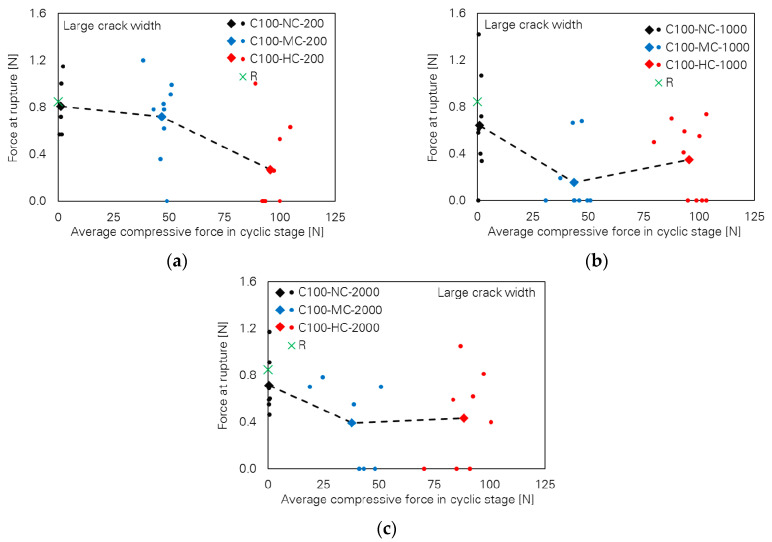
Influence of cyclic compressive force level on force at rupture of the specimens with large crack width; number of cycles: (**a**) 200, (**b**) 1000 and (**c**) 2000.

**Figure 8:**
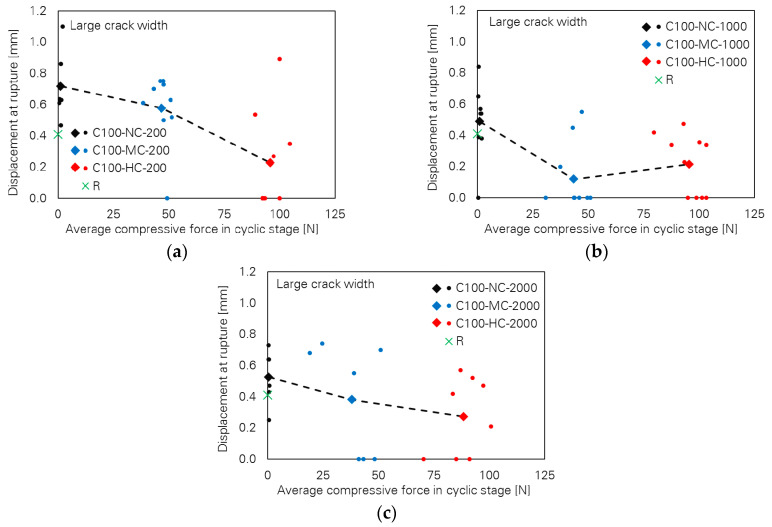
Influence of cyclic compressive force level on fibre displacement at rupture of the specimens with large crack width; number of cycles: (**a**) 200, (**b**) 1000 and (**c**) 2000.

Contrary to observations in cases of small crack width, the influence of the loading parameters on the bridging properties for the specimens with large crack width is very pronounced. This can be seen in Figure 6, where pull-out toughness versus cyclic compressive force level is plotted for different numbers of cycles. A strong decreasing tendency can be observed with increasing cyclic compressive force level. It is noteworthy that many specimens failed in the cyclic stage already; see Table 4, where a classification according to the observed bridging behaviour is given. Thus, pull-out toughness and any other bridging parameter as well assigned to those specimens are zero. The generally decreasing tendency is also the case for the two other bridging parameters, fibre force and fibre displacement at rupture, as illustrated in Figure 7 and Figure 8, respectively.

It is noteworthy that the scattering of the results is considerable in most cases. In general, sources of dispersion could be the different properties of fibres (e.g., fibre diameter) and interface to the matrix (such as distribution of imperfections in the fibre tunnel); see e.g., [30] for further explanation. Additionally, according to the locking front model [31], the initiation of the damage during bridging action of PVA fibre in the post-debonding regime does not follow any particular scheme, which leads to different bridging behaviours of the fibres. Specifically, in an alternating tension–compression regime, the real crack width in the specimen may not be equal to the nominal value set as loading parameter for testing, because of various possible scenarios of matrix cracking. For further explanations about the latter, see Section 5.3 and Figure 12.

### 5.3. Joint Influence of Crack Width and Loading Parameters

**Figure 9:**
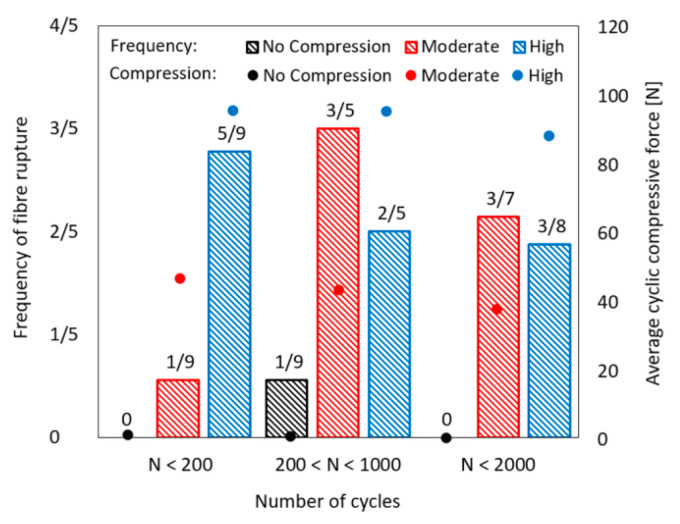
Frequency of fibre rupture during the cyclic stage depending on the number of cycles and compressive force on the fracture section.

The frequency of fibre rupture during the cyclic stage is plotted in Figure 9 for the test series with large crack widths. The results are arranged according to the number of cycles. The average compressive force for individual testing series is also marked in the diagram. The exact numbers of cycles before fibre rupture are given in Table 5 for the corresponding specimens. It can be clearly seen that the fibre rupture during the cyclic stage is only rarely possible as long as the compressive force on the fracture section is small, whereas the probability of fibre rupture increases considerably when the compressive force level increases to a moderate or high level. It is apparent that a number of cycles as few as 200 with a moderate compressive force component on the fracture section is still tolerable as concerns the probability of fibre rupture during the cyclic stage. By increasing the cyclic compressive force to the highest level, the frequency of fibre rupture in the cyclic stage increases significantly. Fibres ruptured in the cyclic stage in more than 50% of the specimens for C100-HC-200 before 200 cycles were completed; see Table 5.

It is to be noted that the number of cycles before fibre rupture for two specimens in C100-MC-2000 series and one specimen in C100-HC-2000 series was smaller than 1000. For this reason, the matching category of the corresponding specimens was changed with respect to the number of cycles, and these are counted in the testing series C100-MC-1000 and C100-HC-1000, respectively. Considering this, the frequency of fibre rupture during the cyclic stage should actually be highest for the testing series C100-MC-2000 and C100-HC-2000. The overview of these results indicates a critical condition for fibre damage development in an alternating tension–compression regime with large crack widths of almost 200 µm. The fibre might rapidly lose its entire bridging capacity when the cyclic compressive stress acting on the fracture section is roughly 12 MPa. In addition to the cyclic compressive stress level, it seems that the displacement increment, which represents indirectly the increase in the bridging stress in the fibre, plays a role in damage development as well. It is the largest for the testing series with 200 cycles and number of specimens with fibre rupture during the cyclic stage is among the highest for this case. Furthermore, the number of cycles to fibre rupture is lowest for C100-HC-200. It seems that the damage severity to the fibre with a number of cycles larger than 200 and moderate compressive force level was already worse than the critical damage condition since fibre rupture during the cyclic stage occurred in more than half of the specimens tested, with one-way full debonding and slippage behaviour observed. This proportion stays almost the same for high cyclic compressive force level; refer to the explanation at the beginning of this paragraph.

**Figure 10:**
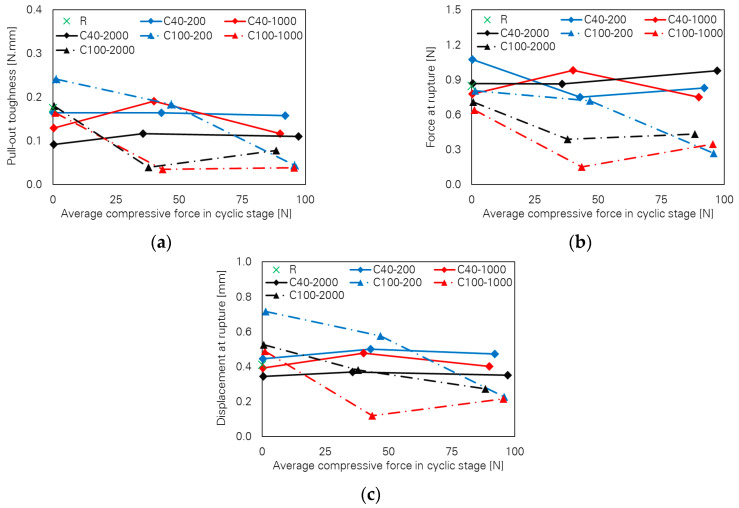
Combined influence of crack width and loading parameters on the crack-bridging capacity of PVA fibres: (**a**) pull-out toughness, (**b**) force and (**c**) displacement at fibre rupture.

A comparison of the bridging parameters as shown in Figure 10 and Table 6 reveals the significance of crack width in development of the fibre’s damage as well as the degradation of its crack-bridging capacity. Clearly, the number of cycles and cyclic compressive force level have major influence on the fibre’s crack-bridging capacity when specimens with the large crack width are considered. In contrast, the influence of these parameters on crack-bridging is negligible in the case of small crack width. Small crack width starts at 40 µm in the beginning of the cyclic stage and reaches 60 µm at its end. In the case of large crack width, it begins at 100 µm and at the end of the cyclic stage lies at 200 µm. The mechanisms responsible for the clear difference between fibre-bridging capacity for small versus large crack widths under an alternating tension–compression regime are summarised in this section.

It is possible that for small crack widths, full debonding does not occur until the end of the cyclic stage with a small number of cycles, where the fibre deforms elastically after partial debonding on both sides of the crack. Thus, the fibre is stretched in the tensile segment of the loading loop and it can deform back into the tunnel in the compressive part of the loading loop. Even if full debonding is considered to occur despite small crack width, the slippage of the fibre is small, leading to a very short damage zone on the fibre between the crack faces, as shown previously [14]. In such a situation, no severe defibrillation of the fibres occurs. Therefore, only slight degradation of the fibre is expected to occur due to the related damage mechanisms in alternating tension–compression regime as introduced in [14].

**Figure 11:**
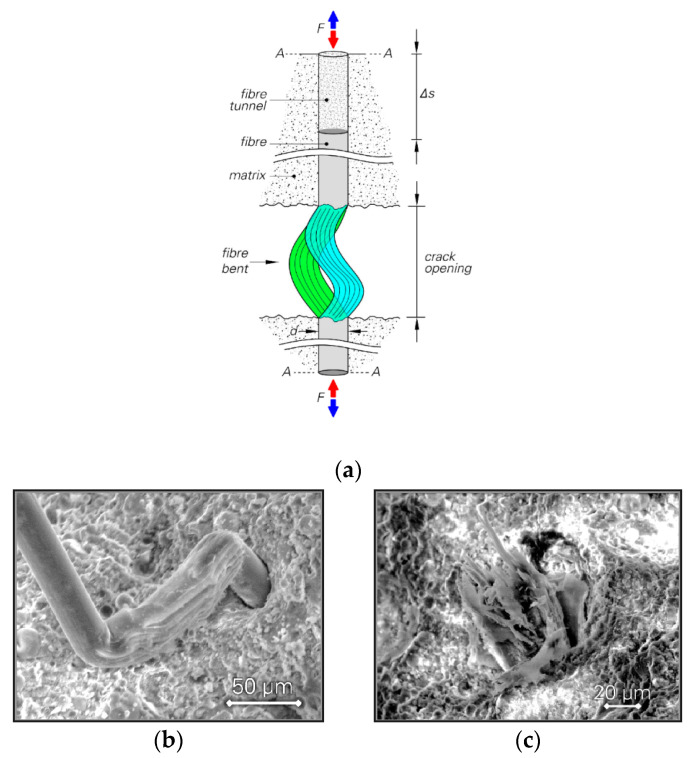
(**a**) Fibre bent due to large crack width under alternating tension–compression loading; scanning electron microscope (SEM) images reveal (**b**) the form of damage for C100-NC-2000-05 and (**c**) ruptured section of the fibre for C100-HC-1000-09.

In cases of large crack width, however, the mechanisms can be markedly different. Firstly, full debonding at least on one embedded side is most probable and fibre slippage is already considerable. There is no lateral support for the fibre between the crack faces. In addition, the fibre might face some obstacles to its being pushed back into the tunnel. The larger the crack width, the higher the probability of the fibre’s bending/buckling in the compressive part of the loading loop; see Figure 11a,b. Even if the crack width is not large enough for bending/buckling of the fibre to occur, the possibility of squeezing/crushing is still high as shown in previous work of the authors [14]. It is noteworthy that due to slip-hardening behaviour, the level of bridging stress in the fibre is likely to be higher for a large crack width than for a small one. Therefore, the development of damage initiated in the compressive part of the loading loop occurs by pulling the fibre in the tensile portion of the loading loop. This may be a partial reason for the rupture of many fibres in the cyclic stage in cases of large crack widths, whereas no fibre rupture was observed during the cyclic stage for the series tested with small crack widths. The anisotropic nature as well as the poor compressive mechanical properties of the fibres in the longitudinal and transverse directions could play a vital role in the unfavourable bridging properties in cases of large crack width. For instance, when the fibre is squeezed between the crack faces, it is no longer straight. Thus, the mechanical properties in the transverse direction are important, particularly as regards compressive behaviour. These damage mechanisms can eventually weaken the fibre between the crack faces and a “fibrillar” [15] fracture type may occur; see also Figure 11c.

Obviously, the average cyclic compressive stress acting on the fracture section is not the most appropriate indicator in considering the detrimental influence of the compressive component on the fibre. The concentrated compressive stress acting on the fibre is limited to a small zone around the fibre tunnel in the fracture region. It depends on the fracture pattern of the matrix and the unevenness of the fracture section and the attitude in which the fibre lies between the crack faces. However, the cyclic compressive stress on the fracture section is the only computable parameter to evaluate the effect of compression on the fibre between crack faces.

**Figure 12:**
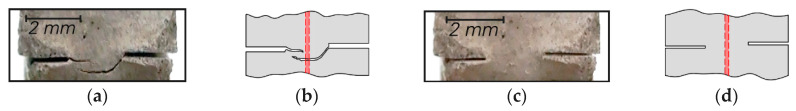
(**a**,**b**) Fracture pattern on the notch at the upper reversal point, (**c**,**d**) the deformed shape of the specimen at the lower reversal point.

A brief comment on the measurement of the crack width is essential here. The displacements in these experiments were measured by the movement of the machine’s crosshead, which provides reliable values of the specimen’s deformations and crack opening displacement, particularly after matrix fracture on the notches, as discussed previously [14,27]. However, real crack opening displacements might be smaller than the values measured using this approach due to the individual fracture pattern. Figure 12a,c shows, for instance, the fracture pattern of a specimen tested in a large crack width in the cyclic stage as the crack opening is the largest on the upper reversal point and it is the smallest on the lower reversal point. For better visibility, the images are redrawn with higher contrast in Figure 12b,d. For this particular specimen the crack opening displacements measured were 200 µm at the upper reversal point by the machine. However, the values of crack opening displacement in the middle of the individual cracks, based on the evaluation of the images are in the range of 40 to 60 µm for two different cracks due to the specific fracture pattern. Thus, it can be concluded that crack width should be measured on the surface of individual specimens, even though only a single crack forms on the specimen and all other deformations, including the deformation of the testing frame and specimen, are negligible after fracturing of the specimen on the notches. This is due to different crack paths’/fracture patterns’ possibly occurring.

## 6. Joint Influence of Crack Width and Loading Parameters

A comparison has been made between the results obtained in the current study and values predicted using the damage quantification approach as presented in [14]. This comparison revealed that the degradation indices lie above the predicted values in the case of small crack widths. In contrast, the predicted degradation indices underestimated the damage for the large crack widths. This suggests that the influence of crack width on degradation indices is very pronounced and as important as the cyclic compressive stress on the fracture surface. Thus, the damage quantification approach needs to be adjusted to consider the crack width. Among the different degradation indices, the index for pull-out force (δ_r_) showed obvious trends depending on crack width and loading parameters. Hence, it has been chosen in adapting the damage quantification approach.

**Figure 13:**
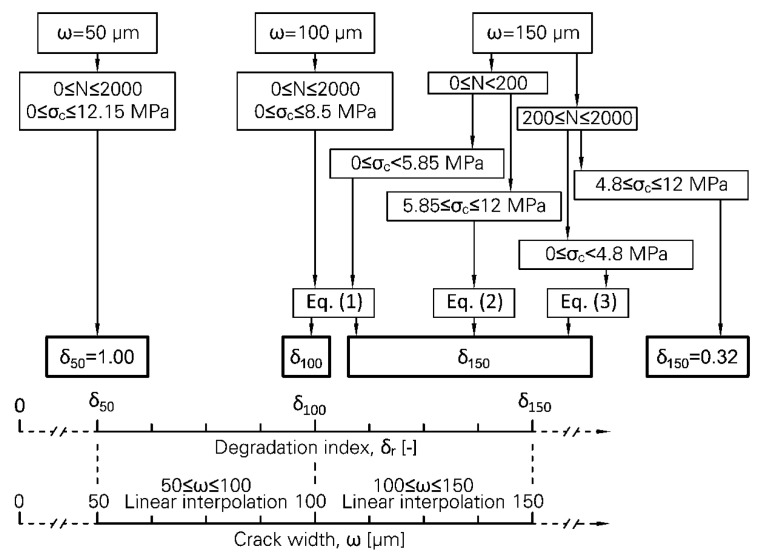
Suggested algorithm for estimation of the crack-bridging capacity of PVA microfibre and its degradation under alternating cyclic loading and under consideration of crack width.

The influence of three parameters, crack width (ω), cyclic compressive stress level on the fracture surface (σ_c_) and number of cycles (N) on fibre degradation in an alternating tension–compression regime has been demonstrated so far. The individual impacts of these parameters on fibre degradation are very similar. However, the development of simple equations for quantifying the degradation depending on these parameters could lead to great inaccuracy when all these parameters are considered to be variable in the equations. Another issue is the large scattering of the results and involvement of coincidental events in fibre pull-out, matrix fracture pattern and fibre degradation and rupture, which make the use of the fitting approach difficult. Hence, an algorithm is employed for adapting the existing damage quantification approach [14] to consider the results of the current investigation. This algorithm is illustrated in Figure 13. The average crack widths of 50 µm and 150 µm in the cyclic stage are considered to represent small and large crack widths, respectively. In the previous study crack width in the cyclic stage was 100 µm for all testing series. Thus, three sets of the results depending on crack width are available with an adequate number of specimens for each case.

To estimate fibre degradation according to the new approach, it is firstly required to choose a value for crack width (ω), which should lie in the intervals 50 µm to 100 µm or 100 µm to 150 µm. Then, depending on the loading parameters, σ_c_ and N, δ_r_ should be estimated for the extreme values in the corresponding interval. Finally, a linear interpolation delivers the target δ_r_ for the chosen crack width. For a crack width smaller than 50 µm, the influence of compressive component of loading on fibre degradation would be negligible for a small number of cycles, i.e., below 2000. It seems that the degradation of the fibre would be more severe for the crack widths larger than 150 µm. However, the use of the new approach is limited to 150 µm based on the results obtained. Equation (1) in Figure 13 is presented in [14]; Equations (2) and (3) are in simple form of δ_r,150_ = 0.0869 σ_c_ + 1.3579 and δ_r,150_ = 0.0955 σ_c_ + 0.8072, respectively.

**Figure 14:**
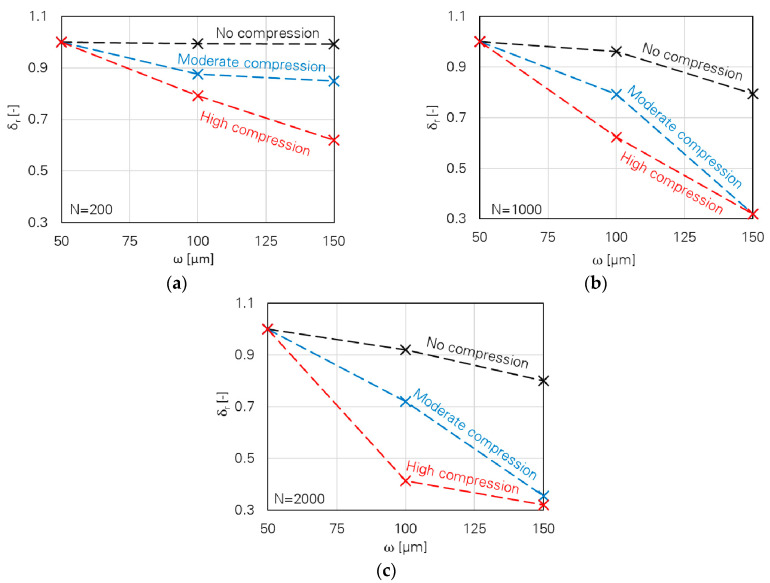
Degradation index (δ_r_) as a function of crack width (ω), cyclic compressive stress (σ_c_) on the fracture surface, and number of cycles (N): (**a**) N = 200, (**b**) N = 1000 and (**c**) N = 2000.

A Python code was developed according to the new approach. The input parameters equal to the average experimental results were put into the programme and the degradation index was calculated. For the case of a high compressive force level, different boundaries for cyclic compressive stress on the fracture surface (σ_c_) are obtained in the current study in comparison to the previous investigation [14]. Thus, this parameter deviates from the average experimental results for small and large crack widths to lie in the valid range as obtained in previous study [14]. The results are displayed in Figure 14; the effects of crack width and the loading parameters are clearly recognisable.

## 7. Conclusions

The results of an extensive investigation on the crack-bridging properties of PVA fibres embedded in a cementitious matrix showed that crack width is an important parameter in an alternating tension–compression regime. While crack widths smaller than 40–60 µm do not affect the degradation of fibres with respect to their mechanical and crack-bridging properties for small numbers of loading cycles i.e., between 200 and 2000, larger crack widths over 100 µm can be detrimental. However, so long as the cyclic compressive stress level on the fracture surface is insignificant, the influence of crack width is negligible for small numbers of cycles as mentioned above. In contrast, the danger of severe damage to the fibres increases with increasing crack width, cyclic compressive stress, and number of cycles. The two former parameters affect the results more markedly. It can be assumed that there is a critical crack width, below which the fibre’s degradation is negligible for small numbers of cycles. It is scarcely possible to determine an exact value of such critical crack width due to the scattering of the results. However, it seems that crack widths below 50 µm are still safe and the critical range should be between 50 and 100 µm, in which the unfavourable influence of the compressive component of cyclic loading on fibre degradation is expected.

The future research at the TU Dresden will focus on the prevention of the fibre damage in an alternating tension–compression loading regime. Obviously, keeping the crack width smaller than the critical value could decrease the damage extent and improve the bridging behaviour of the fibres. This seems to be the most promising solution towards increasing the number of cycles before significant degradation of the bridging capacity of the fibres occurs. Another potential solution could be the use of alternative fibres as already discussed in [32].

## Figures and Tables

**Table 1:** The composition of the matrix (kg/m^3^).

Cement CEM I 42.5 R-HS	Fly Ash (Steament H4)	Water	Quartz Sand (0.06–0.2 mm)	Superplasticizer (Glenium ACE30, BASF)	Viscosity Agent
505	621	338	536	10	4.8

**Table 2:** Physical and mechanical properties of the polyvinyl alcohol (PVA) microfibre.

Fibre Type	Nominal Diameter [µm]	Density [g/cm^3^]	Tensile Strength [MPa]	Young’s Modulus [GPa]	Strain Capacity [-]
Kuralon K-II REC 15 (Kuraray)	40	1.3	1600	40	0.06

**Table 3:** Loading parameters for the testing series under investigation.

Test Series	Crack Width in Cyclic Stage (U_O_) [µm]		Lower Reversal Point (U_U_) [mm]	Displacement Increment Per Cycle (I) [µm]	Number of Cycles [-]	Number of Successfully Tested Specimens
	Start	End				
R	-	-	-	-	-	11
C40-NC-200	40	60	0.020	0.10	200	7
C40-MC-200			−0.010			10
C40-HC-200			−0.025			10
C40-NC-1000			0.020	0.02	1000	4
C40-MC-1000			−0.010			10
C40-HC-1000			−0.025			10
C40-NC-2000			0.020	0.01	2000	10
C40-MC-2000			−0.010			10
C40-HC-2000			−0.025			10
C100-NC-200	100	200	0.020	0.50	200	10
C100-MC-200			−0.010			10
C100-HC-200			−0.025			9
C100-NC-1000			0.020	0.10	1000	11
C100-MC-1000			−0.010			11
C100-HC-1000			−0.025			10
C100-NC-2000			0.020	0.05	2000	10
C100-MC-2000			−0.010			8
C100-HC-2000			−0.025			8

**Table 4:** Summary of tests to study the influence of the crack width under consideration of the observed crack-bridging behaviour.

Series	Number of Specimens				
	Total	Fibre Rupture with One-Way Full Debonding and Slippage	Fibre Rupture with Two-Way Full Debonding and Slippage	Complete Fibre Pull-Out	Fibre Rupture in Cyclic Stage
R	11	9	2	0	-
C40-NC-200	7	6	1	0	0
C40-MC-200	10	10	0	0	0
C40-HC-200	10	6	2	2	0
C40-NC-1000	4	4	0	0	0
C40-MC-1000	10	8	2	0	0
C40-HC-1000	10	8	1	1	0
C40-NC-2000	10	10	0	0	0
C40-MC-2000	10	7	2	1	0
C40-HC-2000	10	10	0	0	0
C100-NC-200	10	6	1	3	0
C100-MC-200	10	9	0	1	1
C100-HC-200	9	9	0	0	5
C100-NC-1000	11	9	0	2	1
C100-MC-1000	11	10	0	1	6
C100-HC-1000	10	10	0	0	4
C100-NC-2000	10	7	3	0	0
C100-MC-2000	8	7	1	0	3
C100-HC-2000	8	8	0	0	3

**Table 5:** Number of cycles before fibre rupture for the specimens with large crack width.

No Compressive Force	Moderate Compressive Force	High Compressive Force
C100-NC-200	C100-MC-200	C100-HC-200
no fibre rupture	162	171 166 129 185 166
C100-NC-1000	C100-MC-1000	C100-HC-1000
752	900 625 937 890 392 573	408 643 653 846
C100-NC-2000	C100-MC-2000	C100-HC-2000
no fibre rupture	1132 1190 1514	1539 1633 1193

**Table 6:** Crack bridging parameters for different series under investigation; average values (standard deviations are given in parentheses).

Test Series	Pull-Out Toughness [N mm]	Force at Fibre Rupture [N]	Displacement at Rupture [N]	Average Maximum Force in Fibre in Cyclic Stage [N]	Average Minimum Force in Fibre in Cyclic Stage [N]
R	0.176 (0.060)	0.847 (0.181)	0.411 (0.088)	-	-
C40-NC-200	0.164 (0.031)	1.075 (0.179)	0.446 (0.037)	0.363 (0.046)	−0.317 (0.177)
C40-MC-200	0.164 (0.045)	0.751 (0.118)	0.502 (0.094)	0.225 (0.056)	−42.928 (6.244)
C40-HC-200	0.158 (0.069)	0.832 (0.302)	0.474 (0.074)	0.157 (0.070)	−92.083 (15.966)
C40-NC-1000	0.130 (0.078)	0.778 (0.245)	0.393 (0.059)	0.168 (0.069)	−0.306 (0.225)
C40-MC-1000	0.191 (0.042)	0.983 (0.134)	0.479 (0.068)	0.201 (0.061)	−40.106 (6.514)
C40-HC-1000	0.117 (0.076)	0.751 (0.153)	0.403 (0.117)	0.152 (0.063)	−89.920 (13.612)
C40-NC-2000	0.092 (0.034)	0.868 (0.171)	0.345 (0.061)	0.246 (0.138)	−0.335 (0.131)
C40-MC-2000	0.117 (0.038)	0.864 (0.157)	0.370 (0.051)	0.151 (0.036)	−35.684 (6.352)
C40-HC-2000	0.110 (0.055)	0.978 0.237	0.351 0.083	0.218 0.106	−97.188 3.202
C100-NC-200	0.242 (0.125)	0.807 (0.214)	0.717 (0.206)	0.260 (0.059)	−1.291 (0.519)
C100-MC-200	0.184 (0.107)	0.719 (0.336)	0.577 (0.222)	0.272 (0.091)	−46.823 (3.795)
C100-HC-200	0.045 (0.070)	0.269 (0.349)	0.227 (0.300)	0.335 (0.100)	−95.733 (4.717)
C100-NC-1000	0.165 (0.143)	0.641 (0.389)	0.488 (0.216)	0.229 (0.069)	−1.045 (0.556)
C100-MC-1000	0.035 (0.071)	0.154 (0.266)	0.120 (0.200)	0.352 (0.087)	−43.544 (5.589)
C100-HC-1000	0.038 (0.036)	0.349 (0.298)	0.216 (0.186)	0.287 (0.070)	−95.496 (7.184)
C100-NC-2000	0.180 (0.122)	0.710 (0.229)	0.526 (0.149)	0.246 (0.069)	−0.510 (0.104)
C100-MC-2000	0.040 (0.037)	0.390 (0.344)	0.381 (0.335)	0.344 (0.062)	−37.954 (11.063)
C100-HC-2000	0.079 (0.092)	0.434 (0.378)	0.274 (0.234)	0.234 (0.043)	−88.367 (8.788)

## References

[1] Mechtcherine V. (2013). Novel cement-based composites for the strengthening and repair of concrete structures. Constr. Build. Mater..

[2] Zhan K., Yu J., Wang Y., Yu K. Development of cementitious composites with tensile strain capacity up to 10%. Proceedings of the International Conference on Strain-Hardening Cement-Based Composites.

[3] Leung C.K.Y. Performance-Based Design of SHCC Components—Research and Challenges. Proceedings of the International Conference on Strain-Hardening Cement-Based Composites.

[4] Leung C.K.Y., Li V.C. (1990). Strength-based and fracture-based approaches in the analysis of fibre debonding. J. Mater. Sci. Lett..

[5] Das A.K., Mishra D.K., Yu J., Leung C.K.Y. (2019). Smart self-healing and self-sensing cementitious composites-recent developments, challenges, and prospects. Adv. Civ. Eng..

[6] Yu J., Yao J., Lin X., Li H., Lam J.Y.K., Leung C.K.Y., Sham I.M.L., Shih K. (2018). Tensile performance of sustainable Strain-Hardening Cementitious Composites with hybrid PVA and recycled PET fibers. Cem. Concr. Res..

[7] Huang B.-T., Wu J., Yu J., Dai J.-G., Leung C. (2020). High-Strength Seawater Sea-sand Engineered Cementitious Composites (SS-ECC): Mechanical Performance and Probabilistic Modeling. Cem. Concr. Compos..

[8] Yu J., Leung C. (2020). Sustainable PVA Fiber-Reinforced Strain-Hardening Cementitious Composites (SHCC) with Ultrahigh-Volume Limestone Calcined Clay. Calcined Clays for Sustainable Concrete.

[9] Lin Z., Kanda T., Li V.C. (1999). On Interface Property Characterization and Performance of Fiber Reinforced Cementitious Composites. Concr. Sci. Eng..

[10] Redon C., Li V.C., Wu C., Hoshiro H., Saito T., Ogawa A. (2001). Measuring and modifying interface properties of PVA fibers in ECC matrix. J. Mater. Civ. Eng..

[11] Kang J., Bolander J.E. Simulating crack width distributions in SHCC under tensile loading. Proceedings of the VIII International conference on fracture mechanics of concrete and concrete structures, FraMCoS-8.

[12] Müller S., Mechtcherine V. (2017). Fatigue behaviour of strain-hardening cement-based composites (SHCC). Cem. Concr. Res..

[13] SASAKAWA E. (1998). A New Synthetic Fiber, KURALON K-II: Its Characteristics and New Applications. Sen’i Gakkaishi.

[14] Ranjbarian M., Mechtcherine V. (2019). Influence of loading parameters in cyclic tension-compression regime on crack-bridging behaviour of PVA microfibres embedded in cement-based matrix. Constr. Build. Mater..

[15] Hearle J.W.S., Lomas B., Cooke W.D. (1998). Atlas of Fibre Fracture and Damage to Textiles.

[16] Van Zijl G.P.A.G., Boshoff W.P., Wagner C., Slowik V. (2017). Introduction: Crack Distribution and Durability of SHCC. A Framework for Durability Design with Strain-Hardening Cement-Based Composites (SHCC).

[17] Li V.C., Wang S., Wu C. (2001). Tensile strain-hardening behavior of polyvinyl alcohol engineered cementitious composite (PVA-ECC). ACI Mater. J. Am. Concr. Inst..

[18] Lepech M., Li V.C. Water permeability of cracked cementitious composites. Proceedings of the ICF 11.

[19] Wang S., Li V.C. Polyvinyl alcohol fiber reinforced engineered cementitious composites: Material design and performances. Proceedings of the Int’l Workshop on HPFRCC Structural Applications.

[20] Wagner C., Slowik V., Waldenburger K. (2008). Dehnungsverfestigendes zementgebundenes Material für die Sanierung gerissener Betonflächen. Bautechnik.

[21] Boshoff W.P., Adendorff C.J. Modelling SHCC cracking for durability. Proceedings of the Fracture and Damage of Advanced Fibre-reinforced Cement-based Materials.

[22] Van Zijl G. Crack distribution characterisation towards a framework for durability design of SHCC. Proceedings of the 2nd International RILEM Conference on Strain Hardening Cementitious Composites (SHCC2-Rio).

[23] Van Zijl G.P.A.G., Slowik V., Toledo Filho R.D., Wittmann F.H., Mihashi H. (2016). Comparative testing of crack formation in strain-hardening cement-based composites (SHCC). Mater. Struct..

[24] Adendorff C.J. (2009). The Time-Dependant Cracking Behaviour of Strain Hardening Cement-Based Composite.

[25] Boshoff W.P., Adendorff C.J. (2013). Effect of sustained tensile loading on SHCC crack widths. Cem. Concr. Compos..

[26] Boshoff W.P. (2014). Cracking behavior of SHCC subjected to sustained tensile loading. ACI Mater. J..

[27] Ranjbarian M., Mechtcherine V. (2018). A novel test setup for the characterization of bridging behaviour of single microfibres embedded in a mineral-based matrix. Cem. Concr. Compos..

[28] Ranjbarian M., Mechtcherine V. Cyclic Damage on PVA Microfibre Embedded in Cementitious Matrix in Alternating Tension-Compression Regime. Proceedings of the FRC2018: Fibre Reinforced Concrete: From Design to Structural Applications Joint ACI-fib-RILEM International Workshop.

[29] Boshoff W.P., Mechtcherine V., van Zijl G.P.A.G. (2009). Characterising the time-dependant behaviour on the single fibre level of SHCC: Part 1: Mechanism of fibre pull-out creep. Cem. Concr. Res..

[30] Jun P., Mechtcherine V. (2010). Behaviour of strain-hardening cement-based composites (SHCC) under monotonic and cyclic tensile loading: Part 1—experimental investigations. Cem. Concr. Compos..

[31] Ranjbarian M., Mechtcherine V., Zhang Z., Curosu I., Storm J., Kaliske M. (2019). Locking Front Model for pull-out behaviour of PVA microfibre embedded in cementitious matrix. Cem. Concr. Compos..

[32] Ranjbarian M., Mechtcherine V. Pilot Investigation into Crack Bridging Behaviour of Different Types of High-Performance Microfibres under Reversed Tension-Compression Loading. Proceedings of the fib Symposium 2019, Concrete-Innovations in Materials, Design and Structures.

